# Atorvastatin treatment is effective when used in combination with mefloquine in an experimental cerebral malaria murine model

**DOI:** 10.1186/1475-2875-11-13

**Published:** 2012-01-10

**Authors:** Jean-Baptiste Souraud, Sébastien Briolant, Jérome Dormoi, Joel Mosnier, Hélène Savini, Eric Baret, Rémy Amalvict, Raoulin Soulard, Christophe Rogier, Bruno Pradines

**Affiliations:** 1 Unité de parasitologie, Unité de recherche sur les maladies infectieuses et transmissibles émergentes - UMR 6236, Institut de recherche biomédicale des armées - antenne de Marseille, Allée du Médecin-colonel Jamot, Parc le Pharo, BP 60109, 13262 Marseille Cedex 7, France; 2 Service d'Anatomie et Cytologie Pathologiques, Hôpital d'Instruction des Armées Saint-Anne, Toulon, France; 3 Service de Biochimie, Hôpital d'Instruction des Armées Laveran, Marseille, France; 4 Unité de Chirurgie et Physiologie Expérimentale, Institut de Recherche Biomédicale des Armées, Marseille, France; 5 Service de Maladies Infectieuses, Hôpital d'Instruction des Armées Laveran, Marseille, France

## Abstract

**Background:**

One of the major complications of *Plasmodium falciparum *infection is cerebral malaria (CM), which causes one million deaths worldwide each year, results in long-term neurological sequelae and the treatment for which is only partially effective. Statins are recognized to have an immunomodulatory action, attenuate sepsis and have a neuroprotective effect. Atorvastatin (AVA) has shown in vitro anti-malarial activity and has improved the activity of mefloquine (MQ) and quinine.

**Methods:**

The efficiency of 40 mg/kg intraperitoneal AVA, alone or in association with MQ, was assessed in an experimental *Plasmodium berghei *ANKA rodent parasite model of CM and performed according to different therapeutic schemes. The effects on experimental CM were assessed through the evaluation of brain histopathological changes and neuronal apoptosis by TUNEL staining.

**Results:**

AVA alone in the therapeutic scheme show no effect on survival, but the prophylactic scheme employing AVA associated with MQ, rather than MQ alone, led to a significant delay in mouse death and had an effect on the onset of CM symptoms and on the level of parasitaemia. Histopathological findings show a correlation between brain lesions and CM onset. A neuronal anti-apoptotic effect of AVA in the AVA + MQ combination was not shown.

**Conclusions:**

The combination of AVA and MQ therapy led to a significant delay in mouse mortality. There were differences in the incidence, time to cerebral malaria and the level of parasitaemia when the drug combination was administered to mice. When used in combination with MQ, AVA had a relevant effect on the in vivo growth inhibition and clinical outcome of *P. berghei *ANKA-infected mice.

## Background

The two major complications of *Plasmodium falciparum *infections are cerebral malaria (CM) and severe anaemia, with an estimated one million deaths worldwide each year, especially in children under five years old in sub-Saharan Africa [1]. Neurological sequelae range from 3-10% in adults, and 25% of child survivors present long-term cognitive impairments [2,3]. CM pathogenesis is multi-factorial and still not fully understood. Post-mortem examination of the brain reveals adherence and sequestration of parasitized red blood cells (pRBC), leucocytes and platelets, followed by plugging of microvessels, necrosis, haemorrhages and oedema [4]. The major hypothesis is that infection triggers the overproduction of pro-inflammatory Th1 cytokines, such as tumour necrosis factor (TNF), interferon-γ (IFN-γ), lymphotoxin (LT) and interleukin-12 (IL-12), leading to the up-regulation of leucocyte adhesion molecules (intercellular adhesion molecule-1 ICAM-1, vascular cell adhesion molecule-1 VCAM-1) on the cerebral microvascular endothelium, sequestration of pRBC, endothelial alterations and vascular obstruction. Additionally, cytokines might modify biochemical cerebral pathways [4-6]. CD8 + and NK/T lymphocyte cytotoxicity and cell trafficking should be also key players in CM pathogenesis [7-9].

Malarial deaths occur, despite effective anti-malarial compounds such as quinine or artemisinin-derivatives combinations. Therefore, a number of adjunct therapies have been proposed, including corticosteroids, osmotic agents, desferrioxamine, exchange transfusion, N-acetyl cysteine, pentoxifylline, hyper immune serum, anti-TNF antibodies, agents for the enhancement of macrophage CD36-mediated phagocytosis of *P. falciparum*, anti-apoptosis agents, heparin, anti-convulsants and erythropoietin, which are based on a different patho-physiological rationale. Only anti-convulsants, exchange blood transfusions, osmotic diuretics and pentoxifylline have been shown to be partially effective [10].

Statins, inhibitors of 3-hydroxy-3-methylglutaryl-Coenzyme A reductase (HMG-CoA reductase), a family of lipid-lowering drugs, have recently been demonstrated to have pleiotropic cholesterol-independent effects. They reduce the "mevalonate pathway", which involves the synthesis of isoprenoids and the intracellular trafficking of membrane-associated proteins such as G-proteins. GTP-binding proteins have crucial roles in intracellular inflammatory signalling by acting as molecular on/off switches for various protein kinases. Although many subfamilies have been described, the farnesylated Ras subfamily and the geranylgeranylated Rho, Rac andCdc42 subfamilies are the most important in the context of sepsis because of their essential role in intracellular inflammatory signalling [11].

Among their pleoiotropic effects, statins are recognized to have the following:

- Anti-inflammatory action; they reduce systemic inflammation by lowering the pro-inflammatory tendencies of macrophages and neutrophils by reducing Th1 and promoting Th2 development [11-13]. The activation of T-lymphocytes and the control of the immune response mediated by major histocompatibility complex class II (MHC-II) and CD40/CD40L are inhibited by statins on endothelial, monocyte and macrophage cells [14-16]. Statins inhibit NK-cell cytotoxicity by interfering with LFA-1-mediated adhesion [17];

- An improvement of endothelial function by modulating nitric oxide synthase (NOS) activity, and consequently, nitric oxide levels by reducing inducible NOS expression and by maintaining or increasing endothelial constitutive NOS production [18-20]. Statins also directly decrease the expression of endothelial adhesion molecules, such as ICAM-1, VCAM-1 and E-selectin [21,22]; moreover, they can prevent *P. falciparum *cytoadherence and endothelial damage [23];

- A beneficial effect on the outcome of infection and attenuating sepsis [11,24,25];

- A neuroprotective effect in the setting of cerebral ischemia [26].

Cerebral malaria shares common pathophysiological features with sepsis, especially with regard to the pathology of the endothelium [27].

Of the different statins, atorvastatin (AVA) showed an in vitro anti-malarial activity even though the presence of an HMG-CoA reductase homologue was not revealed by BLASTX comparison of the *P. falciparum *genome against other protozoal HMG-CoA reductase protein sequences [28,29]. Nevertheless, AVA, used alone at 20 mg/kg of body weight, failed to prevent death from cerebral malaria or to affect the parasitaemia of infected mice [30]. Moreover, AVA improved the in vitro activity of MQ [31], quinine [32] or dihydroartemisinin [33] at the plasma concentrations expected in clinical observations in patients taking 80 mg of AVA daily (0.1 to 0.5 μM) [34].

The different mechanisms of AVA as immunomodulator, neuroprotector and potentialisator of anti-malarial drugs call for in vivo evaluation. Animal models do not exactly reproduce human malaria, but they nevertheless exhibit some similarities to human CM, and the use of the *Plasmodium berghei *ANKA rodent parasite model is generally accepted as one of the valid models for studying experimental cerebral malaria (ECM) pathogenesis [4,35].

The aim of the present work was to test AVA efficacy in a murine model on parasitaemia or death (ECM or severe anaemia) and define schemes of drug use in prophylaxis, alone or in combination. For prophylaxis strategy, mefloquine was chosen as anti-malarial drug. The effects on ECM were determined through the evaluation of brain histopathological changes and neuronal apoptosis. The efficacy of AVA used alone in a therapeutic strategy was evaluated to confirm previous studies.

## Methods

### Mice and ECM

A total of 211 female CBA/J mice, 6-7 weeks old and weighing 18-22 g, from Charles River (France), were used in five independent experiments: 128 for the survival studies and 83 for organ sampling and histological analysis. All animals were pathogen-free and were housed under standard conditions, with unlimited access to food and water. All experiments adhered to French guidelines for animal research and were approved by the ethical committee of the Institut de Recherche Biomédicale des Armées-Antenne de Marseille (Number 2007-02). All efforts were made to minimize animal suffering. Mice were infected on day 0 (D0) with *P. berghei *ANKA parasites by intraperitoneal (i.p.) inoculation with 10^7 ^pRBC from infected donor CBA/J mice, diluted in normal saline.

### Experimental design for drug use

**Figure 1:**
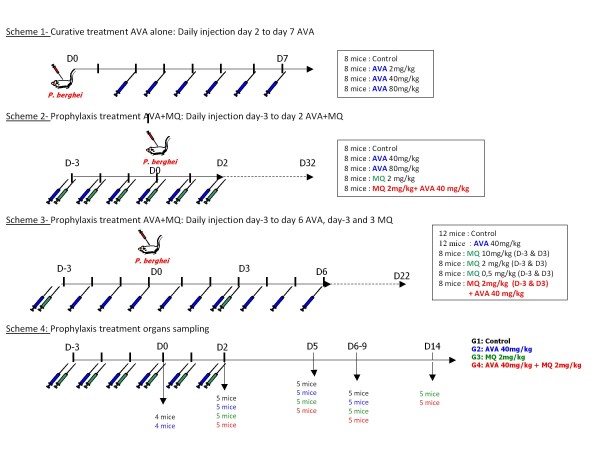
**Experimental design**.

Mice were treated with AVA calcium salt dissolved in 1% dimethyl sulfoxyde (vol/vol) in 0.9% NaCl at various dosages: 2, 40 or 80 mg/kg of body weight; and/or MQ dissolved in 1% methanol (vol/vol) in 0.9% NaCl at various dosages: 0.5, 2 or 10 mg/kg of body weight. Control mice (CT) were treated with NaCl (0.9%) only. Treatment was injected in 0.4 ml by i.p. inoculation using different schemes (Figure 1).

Curative treatment was AVA alone with i.p. injections in four groups of eight mice from D2 to D7: control, 2 mg/kg AVA, 40 mg/kg AVA and 80 mg/kg AVA, respectively (Figure 1, scheme 1).

Prophylaxis treatment was AVA and/or MQ with i.p. injections in five groups of eight mice from D-3 to D2: control, 40 mg/kg AVA, 80 mg/kg AVA, 2 mg/kg MQ and 2 mg/kg MQ associated with 40 mg/kg AVA, respectively (Figure 1, scheme 2).

Prophylaxis treatment was AVA from D-3 to D6 and/or MQ D-3 and D3 with i.p. injections in six groups: control, 40 mg/kg AVA, 10 mg/kg MQ, 2 mg/kg MQ, 0.5 mg/kg MQ and 2 mg/kg MQ associated with 40 mg/kg AVA, respectively (Figure 1, scheme 3). Survival studies were terminated by an animal's death or by killing animals that presented clear signs of CM.

Control, 40 mg/kg AVA, 2 mg/kg MQ and 2 mg/kg MQ associated with 40 mg/kg AVA groups were assessed for histological samples (Figure 1, scheme 4) according to Figure 1, scheme 2, i.e., prophylaxis treatment with AVA and/or MQ with i.p. injections from D-3 to D2. Blood, brain, lung, and spleen samples were taken systematically in each group on D0, D2, and D5; in pre-moribund mice with ECM signs on D6-9 in the control group matched with mice of other groups, on D14 matched with combination group and according to the results of Figure 1, scheme 2 experiments.

### Parasitaemia and clinical parameters

Parasitaemia was determined daily using Giemsa-stained thin blood smears collected from the tailed vein, as the number of infected red blood cells per 3,000 erythrocytes if > 1% and per 10,000 erythrocytes if < 1%. The animals were under daily supervision for clinical signs, neurological symptoms and weight.

ECM was diagnosed by clinical signs based on a simplified SHIRPA protocol [36] with at least two symptoms in at least two of the three different groups:

1. Alteration of autonomous function (piloerection, defecation, urination, respiration rate),

2. Alteration of muscle tone and strength (grip strength, body tone, limb tone, abdominal tone),

3. Ataxia, paralysis (mono-, hemi-, para-, or tetraplegia), deviation of the head, convulsions and coma.

### Organ sampling and histology

Organ sampling was performed according to Figure 1, scheme 4 systematically or in pre-moribund mice with ECM signs. Brains were carefully removed, and cerebral hemispheres were separated: one was frozen at -20°C in RNALater (Qiagen, Germany) for further RNA analysis, and the other one was fixed by immersion in 4% paraformaldehyde with alcohol and acetic acid (AFA) for 48 h. Hemispheres were cut in five coronal sections (rhinencephalon, striatum, hippocampus, posterior thalamus nuclei and cerebellum) [36], embedded in paraffin and serial sections (three at 5 μm), two of which were stained routinely with haematoxylin-eosin-saffron and one that was used for in situ DNA fragmentation detection. Sections were examined by light microscopy, and pictures were taken using a digital camera (Digital Camera DXM1200, Nikon).

### *In situ *detection of DNA fragmentation

Terminal deoxynucleotidyl transferase (TdT)-mediated deoxyuridine triphosphate (dUTP)-digoxigenin nick end labelling (TUNEL) staining was performed according to the manufacturers' protocol, using the TdT-FragEL DNA Fragmentation Detection Kit (Oncogene Research Products, Cambridge, UK). DAB was used as a chromogen, and the sections were counterstained with methyl green. Cellular counting was carried out in a randomized manner by a single investigator. TUNEL + cells were counted in the five coronal sections of one hemisphere.

### Statistical analysis

All statistical analyses were realised with R software (version 2.4.1). Survival analyses were performed by the Kaplan-Meier log rank test. Box plot graphs outlined the 25th and 75th percentiles and the median, with bars representing the minimum and maximum. Box plot graphs were realised with GraphPad Prism 5 software. Parasitaemia were compared by Fisher's exact test. Comparison of medians between multiple groups were analysed by the Kruskal-Wallis test. Comparison of medians between two groups were analysed by the Mann-Whitney test. A difference was considered significant for *P*-values < 0.05.

## Results

### AVA effects on mortality and parasitaemia in ECM

#### Experimental scheme 1, Figure 1

In the curative treatment with injection of AVA alone from D2 to D7, there was no significant difference (*p *= 0.72) in survival between untreated mice and mice treated with a high dose (80 mg/kg), middle dose (40 mg/kg) or low dose (2 mg/kg); nor was there any significant effect on the evolution of parasitaemia.

#### Experimental scheme 2, Figure 1

**Figure 2:**
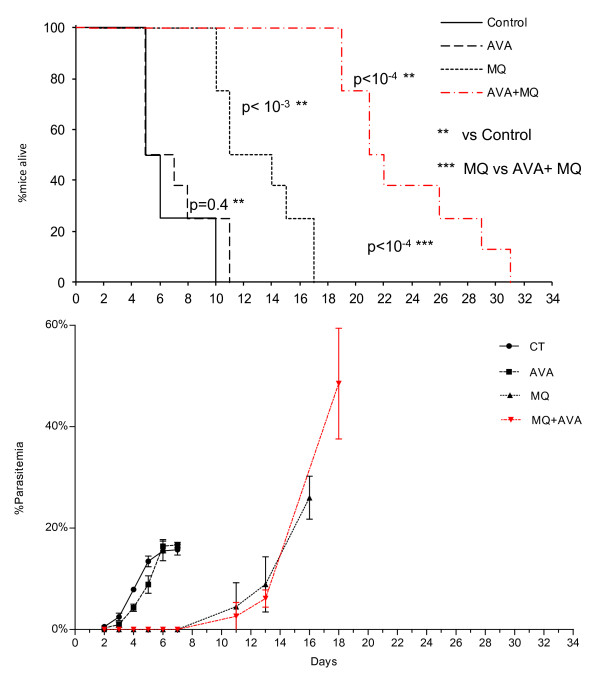
**Survival and parasitaemia level in CBA/J mice infected with PbA and treated with AVA, MQ or AVA + MQ according to Figure 1, scheme 2**.

In this first prophylaxis experiment, 80% of the mice of the control group (CT) and the AVA treated group died with ECM specific signs between D6 and D9 with less than 30% parasitaemia (19.5 to 27%). All of the mice of the two groups died before D12. There was no significant difference between these two groups (*p *= 0.403). Fifty percent of the mice treated with 2 mg/kg of MQ died, some of them exhibiting signs of ECM (2/4), between D11 and D14, and all died before D18 with less than 30% parasitaemia. Survival was significantly different between CT- and MQ-treated mice (*p *< 0.001). Mice treated with the combination of AVA and MQ died between D19 and D32 without signs of ECM. Parasitaemia in this group was greater than 60%. Survival was significantly prolonged in mice treated with the combination (*p *< 0.001) compared to the CT group and to the group treated with MQ alone (*p *< 0.001) (Figure 2).

#### Experimental scheme 3, Figure 1

**Figure 3:**
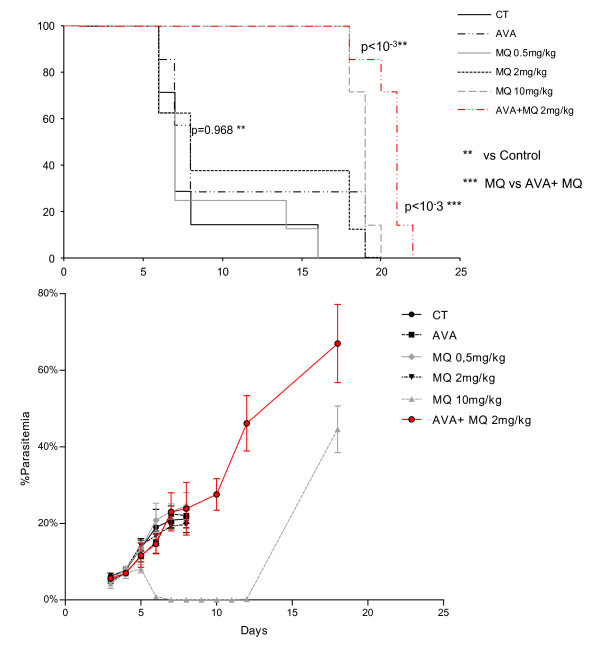
**Survival and parasitaemia level in CBA/J mice infected with PbA and treated with AVA, MQ or AVA + MQ according to Figure 1, scheme 3**.

To confirm the effects of the AVA and MQ combination, another treatment scheme was evaluated with a prophylactic MQ administration identical to once-weekly human prophylaxis. As the recommended prophylactic dose of MQ is 3 to 5 mg/kg, three different doses were tested: 0.5 mg/kg, 2 mg/kg and 10 mg/kg. A combination AVA and MQ dose was 40 mg/kg AVA and 2 mg/kg MQ. There was no statistical difference in parasitaemia evolution between the CT group and the group treated with AVA alone or mice treated with 0.5 mg/kg or 2 mg/kg MQ. The group treated with 10 mg/kg MQ showed a decrease in parasitaemia from 8.1% on D5 to 0% on D7 until D12 and then a rapid increase to greater than 40% on D18, with the death of all mice between D18 and D20. In the group treated with the combination of AVA and MQ, parasitaemia increased to 60% on D18, and all mice died between D18 and D22. There was no significant difference in survival between untreated mice and mice treated with 2 mg/kg MQ (*p *= 0.968). Survival was statistically higher in the mice treated with the combination of AVA and 2 mg/kg MQ compared to the two previous groups, the CT group (*p *< 0.001) and the 2 mg/kg MQ group (*p *= 0.001) (Figure 3).

#### Histological analysis

**Figure 4:**
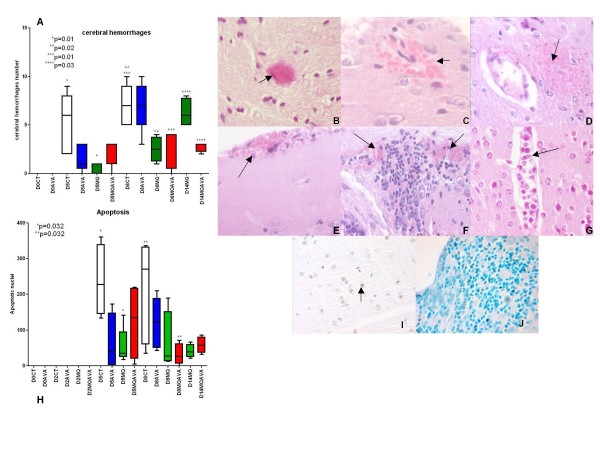
**Histological and TUNEL analysis of mouse brains, sampled according to Figure 1, scheme 4**. (**A**) Number of perivascular and interstitial haemorrhages in two 5 μm serial sections per mouse, in each group from Figure 1, scheme 4. (D0CT = control group at day 0; D0AVA = group with atorvastatin at day 0; D5CT = control group at day 5; D5AVA = group with atorvastatin at day 5; D5MQ = group with mefloquine at day 5; D5MAAVA = group with MQ + AVA at day 5; D8CT = control group at day 8; D8AVA = group with atorvastatin at day 8; D8MQ = group with mefloquine at day 8; D8MQAVA = group with MQ + AVA at day 8; D14MQ = group with mefloquine at day 14; D14MQAVA = group with MQ + AVA at day 14). Significant differences between D5CT and D5MQ, D8CT and D8MQ, D8CT and D8MQAVA, and D14MQ and D14MQAVA. (**B**) HES staining. Petechial haemorrhage 400×. (**C**) Perivascular haemorrhage 1000×. (**D**) Interstitial haemorrhage area with haemozoin pigment 400×. (**E**) Subarachnoid haemorrhage. (**F**) Cerebellum perivascular and interstitial haemorrhage 400×. (**G**) Vascular plugging by monocytes/macrophages (sometimes pigment-containing) with perivascular oedema and few extravasated mononuclear cells 1,000×. (**H**) Number of apoptotic nuclei in one section per mouse from each group of Figure 1, scheme 4; TUNEL + staining (brown colour with DAB). (**I**) TUNEL + neurons revealing apoptotic nuclei; 1000×. (**J**) Focal cerebellar area stained with TUNEL + and showing an interstitial haemorrhage (arrow); 200×. (D0CT = control group at day 0; D0AVA = group with atorvastatin at day 0; D2CT = control group at day 2; D2AVA = group with atorvastatin at day 2; D2MQ = group with mefloquine at day 2; D2MQAVA = group with MQ + AVA at day 2; D5CT = control group at day 5; D5AVA = group with atorvastatin at day 5; D5MA = group with mefloquine at day 5; D5MQAVA = group with MQ + AVA at day 5; D8CT = control group at day 8; D8AVA = group with atorvastatin at day 8; D8MQ = group with mefloquine at day 8; D8MQAVA = group with MQ + AVA at day 8; D14MQ = group with mefloquine at day 14; D14MQAVA = group with MQ + AVA at day 14).

Histological analysis was done on all brains from D0 to D14, and all samples were taken during the Figure 1, scheme 4 experiment. Only CT group mice presented CM signs at D5 (4/5), but all mice in the CT and AVA groups exhibited CM-specific signs at D8; no mouse developed CM signs in the other groups. Only one (1/4) developed CM symptoms at D14 in the MQ group. Qualitative histopathological findings included parenchymal haemorrhages with petechial (Figure 4B), perivascular (Figure 4C) or laminated with occasional haemozoin pigment (Figure 4D), microhaemorrhagic areas, frequently in the cerebellum (Figure 4F), subarachnoid haemorrhages (Figure 4E), perivascular oedema with few extravasated mononuclear cells and vascular plugging by pigment-containing monocytes/macrophages (Figure 4G). These mononuclear cells adhered to endothelial cells, and sometimes small and activated lymphocytes were observed. Vascular plugging and adherence of monocytes to endothelial cells were intense in all animals with CM at D8.

No histological lesions were detected in non-infected mice at D0. Increasing numbers of ring haemorrhages were observed at D5 with a significant difference between the CT group and the MQ group (*p *= 0.01); clinical signs of CM appeared at D5 in the control group. Focal haemorrhages continued to rise at D8 in CT and AVA group, whereas they stayed similar in the MQ and AVA + MQ groups (*p *= 0.02 and *p *= 0.01, respectively). A significant difference was observed at D14 with the increase of haemorrhages in the MQ group (*p *= 0.03) (Figure 4A).

#### Apoptotic neurons analysis

The analysis of apoptosis of neurones was performed on the same samples used for histology analysis. TUNEL + cells showed a tendency to cluster but were sometimes isolated. The number of apoptotic neurons appeared to be increased at D5, with a significant difference between the CT and MQ groups (*p *= 0.032). At D8, TUNEL + cells were less numerous but were significantly lower in the AVA + MQ group, with a median around 25 apoptotic cells per slide, compared to the CT group, which had a median around 270 apoptotic cells per slide (*p *= 0.032). No difference was observed between the two groups remaining at D14 (Figure 4H-J).

## Discussion

These results confirm that in the experimental cerebral malaria model, AVA (used alone) in a therapeutic i.p. treatment shows no effect on the incidence of cerebral malaria or parasitaemia, as previously described [37]. Several statins, such as simvastatin, pravastatin and fluvastatin, used alone are ineffective in cerebral malaria and have no effect on the incidence of parasitaemia during experimental malaria [30,37,38]. In the prophylactic scheme, AVA associated with MQ versus MQ alone leads to a significant delay in mouse death and has an effect on the onset of CM symptoms and on the level of parasitaemia. These experimental conditions did not prevent death, but it appears to be that mice of the combination group did not die of cerebral malaria, as the mice in the three other groups did. Instead, they died of anaemia, with parasitaemia > 60%. Treatment in the MQ group delayed the evolution of parasites, but at the end of the therapeutic effect, parasitaemia increased and CM symptoms appeared.

Experimental models cannot reproduce all the features of human diseases. Differences from the human disease include that leucocytes rather than pRBC are the main cells sequestered in brain vessels, that there are no knobs on pRBC and mice do not develop a high fever [4]. However, the model based on CBA/J mice infected by PbA exhibits primary features also seen in human CM: a clinical picture including coma, seizures and neurological impairment, strong histopathological similarities with impairment of the blood brain barrier, petechial bleedings and systemic and local immune responses involving TNF, INF-γ and IL-1β as pro-inflammatory cytokines [5,35,37,39]. In the present study, as is common, death occurs in six-10 days and involves 80 to 90% of the mice. Different host-parasite factors such genetic background, age, amount of inoculum, course of parasitaemia and clonal variations of the parasite may also interfere with the incidence of CM and may explain the different levels of parasitaemia in our experiments [39].

The major histopathological findings in the brain were petechial haemorrhages predominantly localized in the deep white matter and cerebellum, perivascular oedema and vascular plugging by leucocytes; these changes are similar to those that have been described previously [36,40,41]. However, murine CM does not present reliable histopathological indicators: mice without CM signs may have brain lesions and haemorrhagic areas affecting 0.19% of total cerebral area in mice with clinical signs versus 0.04% in mice without signs [40]; the present data demonstrate a significant increase in brain haemorrhages in mice with CM symptoms. Some studies reported a higher frequency of haemorrhagic foci in the brains of mice showing signs of cerebral involvement when compared with infected animals without clinical manifestation [36,40,41].

Wiese et al. [42] found apoptosis of neurons as well as apoptosis of endothelial cells in the brains of mice with ECM in terminally ill animals, with clear clinical signs of cerebral involvement on days 10-13 after inoculation. Using the same techniques as Wiese, an earlier apoptotic effect was found with an increase in apoptotic neuron nuclei at D5. This may be linked with the high level of parasitaemia in this experiment (at D5: CT 20.8%, AVA 18.6%, MQ 5%, AVA + MQ 9.6%) compared to the parasitaemia level found in Weise's study, at 7.5% for mice with severe symptoms of CM (D10 to D13). The anti-apoptotic effect of AVA in the AVA + MQ combination groups could not be demonstrated here, despite protection against cerebral malaria by the combination. Further studies are needed to confirm the apoptosis effect in AVA treatment.

## Conclusions

Association AVA + MQ is effective in a prophylactic scheme and can potentiate the action of MQ. Immunodulation by AVA is associated with protection against CM. Further immunological study of AVA and AVA + MQ in an ECM model is required and should involve microarray analyses on the brain and cytokine assays. However, effects of AVA + MQ were only evaluated in a prophylactic strategy and not in a treatment strategy. This limits the potential use of AVA. Further studies on the therapeutic treatment scheme are needed to validate AVA as a new partner drug. Resistance to artemisinin-based combination therapy is emerging in southern Cambodia and Thailand, especially the mefloquine-artesunate combination [43]. All these observations support calls for clinical trials of AVA as an anti-malarial therapy.

## Competing interests

The authors declare that they have no competing interests.

## Authors' contributions

JBS, SB, JD conceived, designed, performed the in vivo experiments and drafted the manuscript. JM, EB, RA, HS, NT, RS participated in experiments. BP and CR conceived, designed and coordinated the study and drafted the manuscript. All authors read and approved the final manuscript.
